# Revealing the combined effects of lactulose and probiotic enterococci on the swine faecal microbiota using 454 pyrosequencing

**DOI:** 10.1111/1751-7915.12370

**Published:** 2016-06-16

**Authors:** Jong Pyo Chae, Edward Alain B. Pajarillo, Ju Kyoung Oh, Heebal Kim, Dae‐Kyung Kang

**Affiliations:** ^1^ Department of Animal Resources ScienceDankook UniversityCheonan330‐714Korea; ^2^ Department of Agricultural BiotechnologySeoul National UniversitySeoul151–921Korea

## Abstract

Demand for the development of non‐antibiotic growth promoters in animal production has increased in recent years. This report compared the faecal microbiota of weaned piglets under the administration of a basal diet (CON) or that containing prebiotic lactulose (LAC), probiotic *Enterococcus faecium* NCIMB 11181 (PRO) or their synbiotic combination (SYN). At the phylum level, the *Firmicutes* to *Bacteroidetes* ratio increased in the treatment groups compared with the CON group, and the lowest proportion of *Proteobacteria* was observed in the LAC group. At the family level, *Enterobacteriaceae* decreased in all treatments; more than a 10‐fold reduction was observed in the LAC (0.99%) group compared with the CON group. At the genus level, the highest *Oscillibacter* proportion was detected in PRO, the highest *Clostridium* in LAC and the highest *Lactobacillus* in SYN; the abundance of *Escherichia* was lowest in the LAC group. Clustering in the discriminant analysis of principal components revealed distinct separation of the feeding groups (CON, LAC, PRO and SYN), showing different microbial compositions according to different feed additives or their combination. These results suggest that individual materials and their combination have unique actions and independent mechanisms for changes in the distal gut microbiota.

## Introduction

In the animal industry, much‐improved farming systems and cost‐effective techniques for the production of pathogen‐free high‐quality meat are important goals of current research (Lallès *et al*., 2007; Lee *et al*., 2012; Bomba *et al*., 2014). In recent years, the gastrointestinal (GI) tract and its resident microbiota have been recognized as influential host genetic elements and environmental factors in improving overall animal health, growth and performance (Kim and Isaacson, 2015). The microbial ecosystem of the GI tract plays an important role in preserving a stable and thriving gut environment through its impact on host physiology and functionality (Richards *et al*., 2011), modulation of metabolic activities and immunological responses (Hemarajata and Versalovic, 2012), and provision of a natural defence system against pathogenic invasion (O'Connor *et al*., 2014). Several factors that affect intestinal microbiota in pigs have recently been investigated, including age (Kim *et al*., 2011), diet (Yan *et al*., 2013), weaning (Pajarillo *et al*., 2014a) and antibiotic growth promoters (AGP) (Unno *et al*., 2014). These studies have revealed important concepts in animal farming and management, as well as in the development of non‐AGP.

Pigs are constantly subjected to harsh and stressful conditions during their growth (Lallès *et al*., 2007; Pluske *et al*., 2009; Bomba *et al*., 2014). Previous studies have shown that extreme physiological and morphological changes, including reshaping of the microbiota, occurs in the GI tract of piglets (Lallès *et al*., 2007; Pajarillo *et al*., 2014a). The ban of AGP in feeds in some countries caused serious bacterial and viral infections and increased mortality (Unno *et al*., 2014). Hence, the development of AGP‐alternatives, particularly eubiotics (e.g. probiotics, prebiotics and synbiotics), to boost animal health and enhance growth performance is important. Probiotics and prebiotics can improve the health of their host by balancing the gut microbiota (Hemarajata and Versalovic, 2012; Kim and Isaacson, 2015). Recent studies have shown that performance levels and growth indicators in weaned piglets were significantly increased by probiotic (i.e. lactobacilli) or prebiotic (i.e. inulin, lactulose) administration through increased butyrate supply, heightened villus height for intestinal integrity and better immunomodulation in the gut (Konstantinov *et al*., 2004; Krause *et al*., 2010; Lee *et al*., 2012; Guerra‐Ordaz *et al*., 2014; Sattler *et al*., 2014). Furthermore, the combination of probiotics and prebiotics, called synbiotics, has shown promising results as a non‐AGP (Guerra‐Ordaz *et al*., 2014; Sattler *et al*., 2014). The effects of probiotics and prebiotics in the swine gut microbiota were investigated using both culture‐dependent and ‐independent approaches, such as quantitative PCR and denaturing gradient gel electrophoresis (Lee *et al*., 2012; Martinez *et al*., 2012; Guerra‐Ordaz *et al*., 2014; Sattler *et al*., 2014; Pajarillo *et al*., 2015a). However, previous studies have focused on a handful of microbial groups, typically fewer than 10 bacterial taxa. Using 16S rRNA gene pyrosequencing technology in combination with bioinformatics tools enables more efficient and informative quantification and statistical comparison of microbial diversity and composition across samples.

Recently, independent administration of the probiotic *E. faecium* NCIMB 11181 (Pajarillo *et al*., 2015a) and the prebiotic lactulose (Chae *et al*., 2015) in weaned piglets showed differences in faecal microbial diversity and bacterial community composition. The aim of this study was to examine the synbiotic effect of lactulose and the probiotic bacterium *Enterococcus faecium* NCIMB 11181 on the microbial diversity of weaned piglets and to compare the unexplored synergistic effect against the individual effects of the prebiotic and probiotic on the structure and composition of faecal microbiota, using pyrosequencing of the 16S rRNA genes.

## Results

### DNA sequence data and quality control

Seventy‐nine piglets were divided into four groups: control (CON; *n* = 15), LAC (*n* = 15), PRO (*n* = 20) and SYN (*n* = 29). The pyrosequencing data were generated and pooled for each group. In total, 80 251, 100 172, 98 156 and 208 660 high‐quality sequence reads were obtained in the CON, LAC, PRO and SYN groups respectively. The average numbers of sequence reads generated per pig were 5350, 6678, 4907 and 7195 in the CON, LAC, PRO and SYN groups respectively (Table S1).

### Microbial diversity

**Figure 1:**
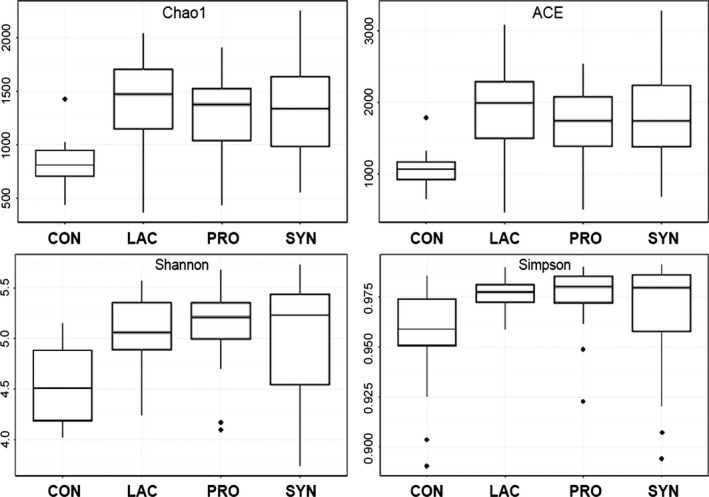
α‐Diversity measurements of pig faecal microbiota according to treatment. Microbial richness estimates (Chao1 and ACE) and diversity indices (Shannon and Simpson) provide measures of diversity within each community at an OTU identity cut‐off of 97%. Each group is labelled accordingly: CON, control; LAC, prebiotic lactulose; PRO, probiotic *Enterococcus faecium* NCIMB 11181; SYN, synbiotic. The Kruskal–Wallis non‐parametric test for significance was performed to assess differences among pig groups; *P*_C__hao1_ = 0.001, *P*_ACE_ = 0.001, *P*_Shannon_ = 0.002, *P*_Simpson_ = 0.03.

α‐diversity measurements comparing the microbial communities of these groups revealed significant differences in piglets among the LAC, PRO and SYN groups compared with the CON group (Fig. 1, Table S1). Rarefaction curves of pooled samples were determined at an operational taxonomic unit (OTU) definition of 97% identity (Fig. S1). The richness estimates, including abundance coverage estimate (ACE) and Chao1 for the LAC, PRO and SYN groups were significantly higher than those for the CON group (*P* < 0.05). The median values for both ACE (1992) and Chao1 (1472) were highest in the LAC group (Table S1), indicating that the microbial communities in piglets that ingested the administered prebiotics exhibited increased numbers of unique species. The Shannon and Simpsonsl (1‐D) diversity indices were also increased by feeding prebiotic lactulose and/or probiotic *E. faecium* NCIMB 11181 (Fig. 1, Table S1). The highest median Shannon value was observed in the SYN group (5.23), and the highest Simpson value was detected in the PRO group (0.980) (Table S1); by contrast, the lowest diversity index was found in the CON group. These diversity indices indicate the number of different bacterial OTUs and populations of microorganisms present in a sample; higher values denote greater diversity. Although the inclusion of prebiotics and/or probiotics in the diet significantly increased α‐diversity compared with CON values, no differences were observed in richness or diversity values among the three treatment groups (LAC, PRO and SYN).

### Comparison of faecal microbial shifts in response to the administration of prebiotics, probiotics and synbiotics: Taxon‐based analysis

**Figure 2:**
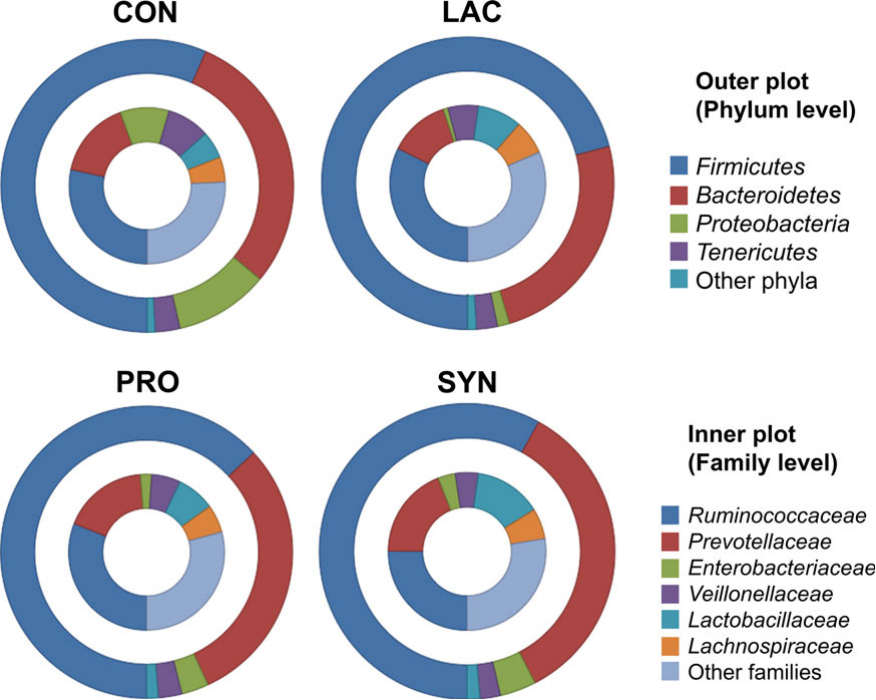
Doughnut plots of the relative abundances of sequences at the phylum and family levels. The EzTaxon database was used to classify the taxon groups. Mean relative abundances were calculated from all samples in each group; outer and inner plots depict selected taxa at the phylum and family levels respectively.

A taxon‐based approach was performed using the EzTaxon database to investigate changes in the composition of the faecal microbiota of weaned piglets after administration of prebiotics, probiotics and synbiotics. The relative abundances at the phylum and family levels are shown in Fig. 2. At the phylum level, the majority of sequences (> 90%) belonged to the *Firmicutes* and *Bacteroidetes*, regardless of the feed additive types. The ratio of *Firmicutes* to *Bacteroidetes* increased in the LAC group (Fig. 2 and Table S2). The abundance of *Proteobacteria* was highest in the CON group, whereas *Proteobacteria* abundances in the LAC, PRO and SYN groups were decreased. The lowest proportion of *Proteobacteria* was found in the LAC group.

At the family level (Fig. 2), the most abundant bacterial groups were *Ruminococcaceae* and *Prevotellaceae* in all pig groups (Table S2), regardless of the treatment. In addition, *Enterobacteriaceae, Veillonellaceae, Lactobacillaceae* and *Lachnospiraceae* were also detected as major bacterial groups (Fig. 2). After administration, the average population of *Lactobacillaceae* was increased by the LAC (9.20%), PRO (7.97%) and SYN (13.8%) treatments compared with the CON (5.67%) group. Additionally, the highest proportion of *Lachnospiraceae* was detected in LAC (7.07%), followed by SYN (6.26%) (Table S2). Furthermore, large decreases in the proportions of *Enterobacteriaceae* were found in all treatment groups; in particular, more than a 10‐fold reduction in LAC (0.99%) was detected compared with the CON group (Fig. 2).

**Figure 3:**
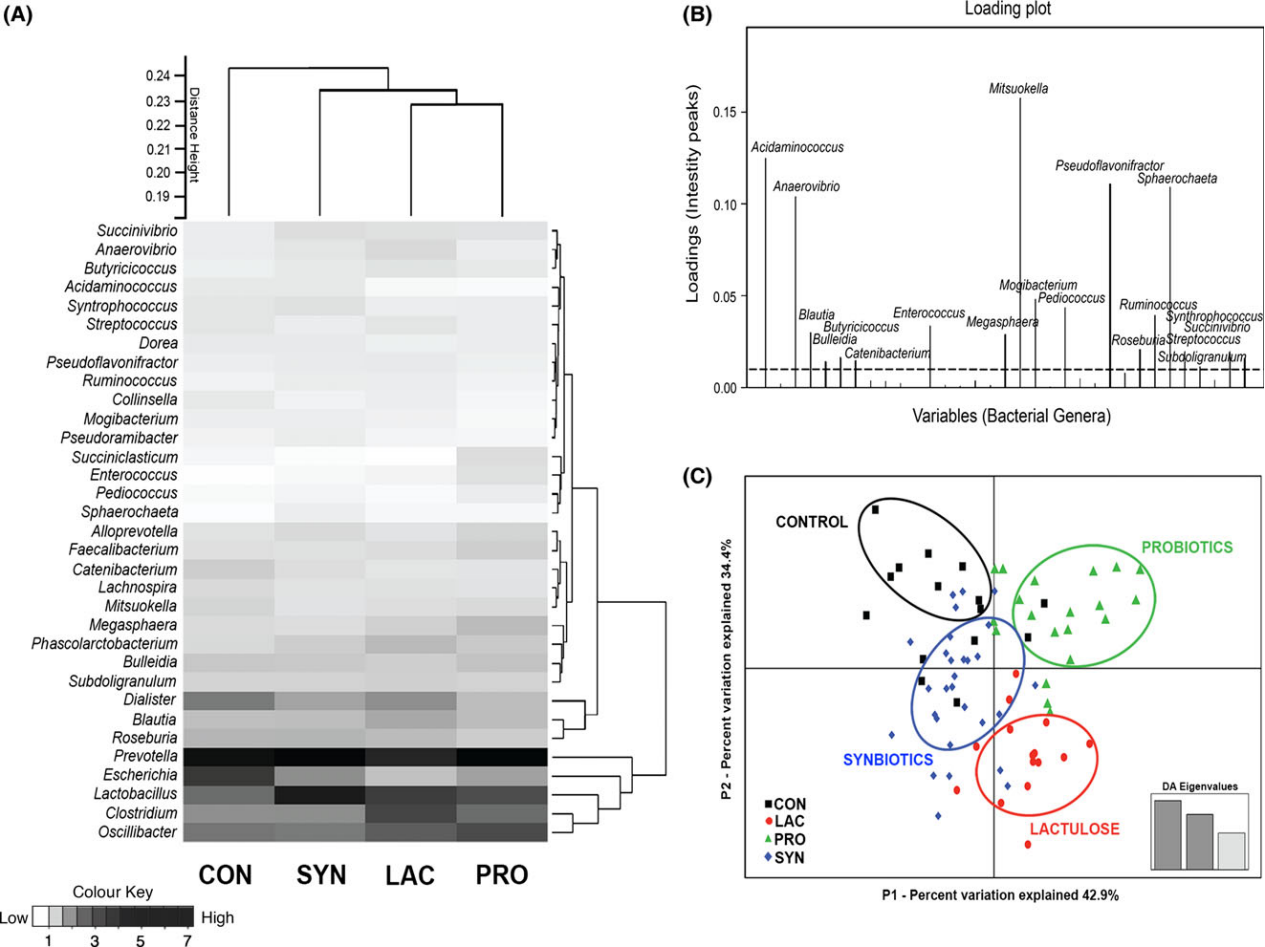
Differentially abundant bacterial genera among the CON, LAC, PRO and SYN groups. Piglets in the control group received a basal diet during the entire experimental period, whereas piglets receiving treatments were fed the basal diet plus the assigned feed additive. A. The heatmap shows the 33 abundant genera (> 0.1% mean relative abundance) after normalization. The normalized levels of abundance are depicted in the colour key, where white represents the lowest (min=0) and black (max=7) shows the highest level of abundance. Columns represent treatment groups, and rows indicate the bacterial genera. B. The canonical loading plot shows peaks for the bacterial genera that had strong influences on the differentiation of the control from the treatment groups. C. Clustering of the faecal microbiota according to treatment was performed by DAPC plot using the 33 differentially abundant bacterial genera as variables.

A total of 99 bacterial genera were identified from at least one faecal microbiota sample in this experiment, including 33 differentially abundant genera (> 0.1% of total sequences) (Fig. 3A). *Prevotella, Lactobacillus, Oscillibacter*,* Clostridium* and *Escherichia* genera were considered more abundant (*x* > 1.0% mean abundance) compared with the remaining 28 bacterial genera (1.0% > *x* > 0.1% mean abundance) (Fig. 3A). Moreover, these five highly abundant bacterial genera were also identified as part of the core microbiota of the swine distal gut in a previous report (Pajarillo *et al*., 2015b). Differential levels of abundance were detected among the feeding groups; the highest proportion of *Oscillibacter* was detected in the PRO group, the highest *Clostridium* in the LAC group, and the highest *Lactobacillus* in the SYN group. The administration of feed additives decreased the number of *Escherichia*, especially in the LAC group.

A dendrogram was constructed using the Bray–Curtis dissimilarity matrix to assess the similarity of the bacterial communities among treatment groups (Fig. 3A). The distinguishing variables (bacterial genera) were plotted as discriminant peaks to determine which of the differentially abundant genera had the greatest influence on the dissimilarity among the CON, LAC, PRO and SYN groups (Fig. 3B). The canonical loading plot displayed the five most influential bacterial genera, namely, *Mitsuokella*,* Acidaminococcus*,* Pseudoflavonifractor*,* Sphaerochaeta* and *Anaerovibrio*. These discriminant peaks for each variable were directly proportional to the strength of influence on differences among groups, with higher peaks depicting stronger influence on the variation and *vice versa*. Next, the discriminant analysis of principal components (DAPC) showed the separation of individual microbial communities in a two‐dimensional plot (Fig. 3C). This plot revealed the significantly separated clustering of pig faecal microbiotas according to treatment group (CON, LAC, PRO and SYN).

### Comparison of faecal microbial shifts in response to the administration of prebiotics, probiotics and synbiotics: OTU‐based analysis

**Figure 4:**
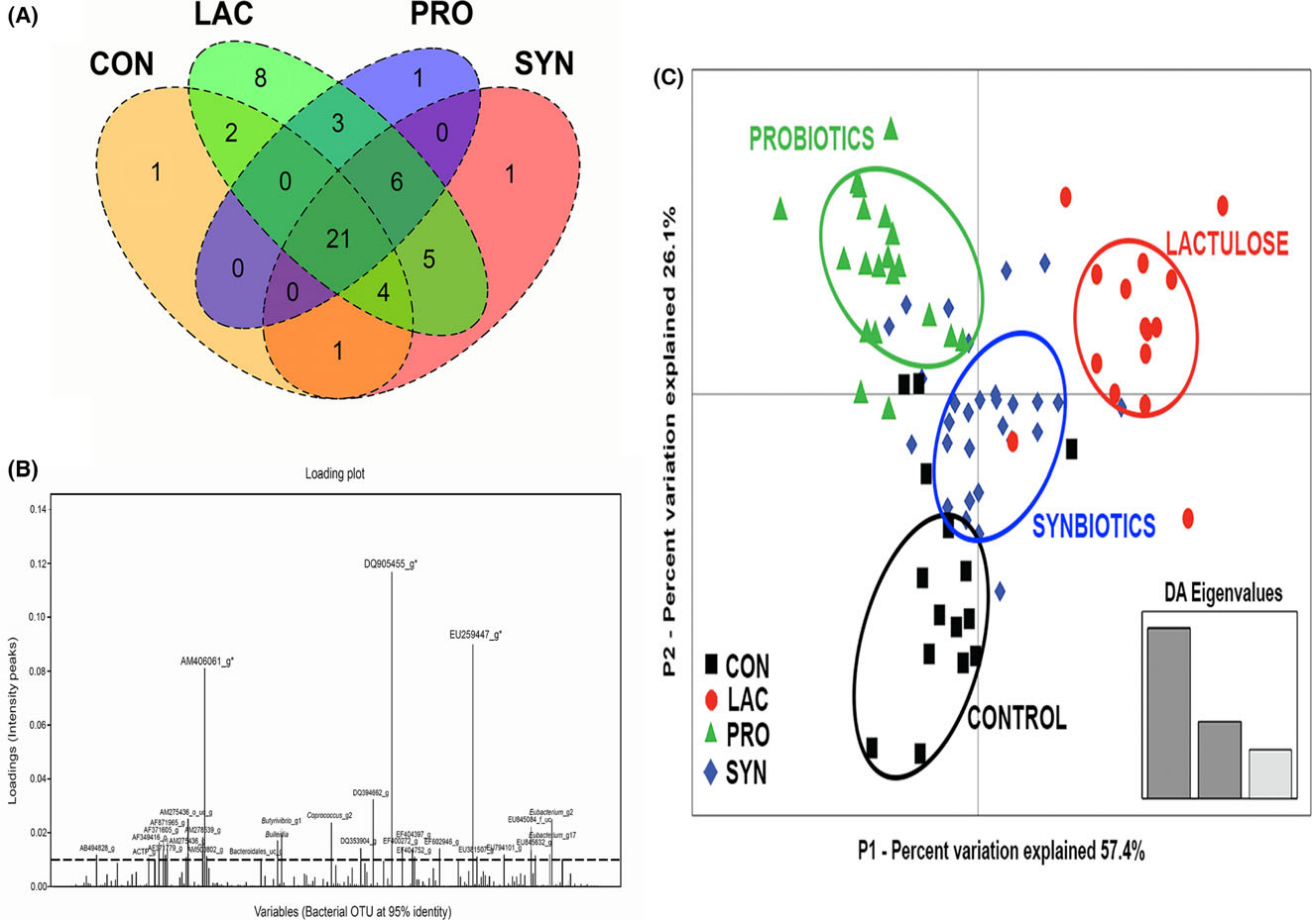
OTU‐based community structures and compositions in the faecal microbiota after treatment. A. Venn diagram showing the distribution of OTUs for the CON, LAC, PRO and SYN groups. Numbers indicate the number of OTUs that were unique and the number shared (core) by two or more groups, as depicted by non‐intersecting and intersecting ellipses respectively. B. The canonical loading plot shows the peaks of all bacterial OTUs that had strong influences on the separation of the control and treatment groups. C. Clustering of the faecal microbiota according to treatment was performed by DAPC plot using all bacterial OTUs at a 95% identity cut‐off as variables.

An OTU‐based approach was conducted for an all‐inclusive membership analysis and in‐depth ecological investigation of the bacterial communities under the influence of prebiotics, probiotics and synbiotics. The OTUs used in this analysis were defined at 95% sequence identity. In all, 397 bacterial OTUs were identified in at least one pig; 253, 287, 335 and 338 OTUs were detected in the CON, LAC, PRO and SYN groups respectively. Next, a Venn diagram was created to describe the core (shared) and unique (distinct) bacterial OTUs among pig groups (Fig. 4A). The overlap of two or more ellipses denotes the shared bacterial OTUs between two or more pig groups. Of the 397 OTUs, 21 were considered to be core (shared) OTUs. In terms of abundance, the core microbiota accounted for more than 50% of the total bacterial population (percent abundance) in the swine distal gut; however, the core only accounted for 5.2% of all bacterial phylotypes (21 of 397 bacterial OTUs) detected in at least one pig sample.

The canonical loading plot was applied to the 397 bacterial OTUs to determine the most influential bacterial phylotypes after the administration of feed additives. According to the EzTaxon database, the three bacterial OTUs that had the highest peaks were DQ905455_g, AM406061_g and EU259447_g; the two former bacterial phylotypes were also found in the core microbiota, whereas the last (EU259447_g) was present at a higher abundance in the faecal microbiota of pigs belonging to the LAC group (Fig. S2). However, other bacterial OTUs showed relatively higher peaks that may help discriminate among the pig treatment groups (Fig. 4B). The DAPC clustering of the faecal microbiotas according to treatment group illustrates the distinct separation of the feeding groups (CON, LAC, PRO and SYN), showing that microbial communities have different compositions according to the type of feed additives (Fig. 4C).

## Discussion

Prebiotics, probiotics and synbiotics have beneficial effects on animal health and nutrition (Krause *et al*., 2010; Martinez *et al*., 2012; Guerra‐Ordaz *et al*., 2014; Sattler *et al*., 2014; Umu *et al*., 2015). The administration of the probiotic *E. faecium* NCIMB 11181 or the prebiotic lactulose, which are used to improve animal health and performance, showed significant shifts in the swine faecal microbiota (Chae *et al*., 2015; Pajarillo *et al*., 2015a). In this study, the synbiotic effects of the lactulose and *E. faecium* NCIMB 11181 combination were compared with the effects of probiotic or prebiotic administration on the overall microbial diversity and bacterial composition of swine faeces using a high‐throughput pyrosequencing method.

First, significant shifts in the structure and proportion of specific bacterial phyla, families, genera and OTUs were detected in the CON, LAC, PRO and SYN piglets. Although *Firmicutes* and *Bacteroidetes* remained the most dominant bacterial groups regardless of treatment, the ratio of *Firmicutes* to *Bacteroidetes* significantly increased in the LAC compared with the CON group. The increase in the *Firmicutes* to *Bacteroidetes* ratio in the LAC group suggests that lactulose promotes the proliferation of some groups in the *Firmicutes* phylum, which may promote various metabolic activities and fermentation of complex plant‐based diets (Hooda *et al*., 2012; Sattler *et al*., 2014; Umu *et al*., 2015). Several studies show that lactulose supplementation improves growth performance, short‐chain fatty acid composition and microbial populations in swine and poultry (Fleige *et al*., 2007; Cho and Kim, 2013; Zheng *et al*., 2014a). The effect of lactulose in the gut increases bacterial diversity and stimulates the growth of many bacteria belonging to *Firmicutes*, including lactobacilli and clostridia (Konstantinov *et al*., 2004; Mao *et al*., 2014). Lactulose‐utilizing bacteria contribute to short chain fatty acids (SCFA) and equol production, which may induce anti‐inflammatory and antioxidant properties in swine intestines (Ito *et al*., 1997; Zheng *et al*., 2014b; Ziar *et al*., 2014). In addition, the higher ratio of *Firmicutes* to *Bacteroidetes* in younger piglets may be advantageous for increasing intestinal SCFA and reducing infection (Molist *et al*., 2012), which is also correlated with increasing body weight (Guo *et al*., 2008).

At the family level, the SYN group had the highest abundance of *Lactobacillaceae*, which suggests complementary effects between the prebiotic oligosaccharide and probiotic bacterium. The *Lactobacillaceae* family comprises well‐known probiotic bacteria that are generally recognized as safe and are highly adapted to the GI environment (Etzold *et al*., 2014), which improve overall GI integrity and functionality. This family is described as the energy‐generating machinery in humans and animals by increasing the levels of short‐chain fatty acids, particularly acetate, propionate and butyrate, in the gut (Hooda *et al*., 2012; Guerra‐Ordaz *et al*., 2014). In a previous study, the synbiotic mixture of *L. plantarum* and lactulose produced different effects on the microbial populations in pigs (Guerra‐Ordaz *et al*., 2014). Consequently, the combination of *E. faecium* NCIMB 11181 and lactulose in this study suggests the formation of unique metabolites or compounds that trigger the growth of *Lactobacillaceae*. Decreased abundances of *Enterobacteriaceae* and *Veillonellaceae* families were also observed in all treatment groups. Low populations of *Enterobacteriaceae* are favourable for animal production, because increases in this family are likely associated with high mortality in pigs, caused by bacterial infection (Pluske *et al*., 2009; Krause *et al*., 2010). Reduction in the abundance of *Veillonellaceae* may also have positive effects on pig health, due to its association with nasopharyngeal infections and GI‐associated diseases, as well as with cirrhosis and extreme levels of bile acids in the gut (Bajaj *et al*., 2011; Gevers *et al*., 2014).

At the genus level, the abundances of many bacterial genera were affected. First, the genus *Lactobacillus* increased in abundance in all treatment groups, which was notably highest in the SYN group. Lactobacilli are responsible for higher levels of anti‐inflammatory and systemic responses and for out‐competing and exclusively displacing pathogenic bacteria along the mucosal surfaces of the host (Etzold *et al*., 2014; Johnson and Klaenhammer, 2014). However, the synbiotic effect on the increased lactobacilli population may be dependent on the specific probiotics and prebiotics used, as well as their dosages, because the synbiotic effect of *L. plantarum* and lactulose in the *Lactobacillus* population did not exceed the individual effects of singular administration of the prebiotic or probiotic (Guerra‐Ordaz *et al*., 2014). On the other hand, the number of *Escherichia* decreased sharply in all treatment groups, most remarkably in the LAC group. Previous reports showed that the lactulose effect was most distinguishable on the population of *Escherichia*, specifically enterotoxigenic and enteropathogenic *E. coli* K88 at post‐weaning (Konstantinov *et al*., 2004; Krause *et al*., 2010; Guerra‐Ordaz *et al*., 2014).

Furthermore, the identified discriminating bacterial genera may have contributions to gut functions in relation to the feed additives with which they are associated. Investigating the influence of probiotic and prebiotic interventions not only in a handful of bacterial groups, which was common in previous studies (Guerra‐Ordaz *et al*., 2014; Zheng *et al*., 2014a), but on the total gut microbiota revealed unique bacterial genera that may be beneficial or harmful to pigs; specifically *Mitsuokella*,* Acidaminococcus*,* Pseudoflavonifractor, Sphaerochaeta*, and *Anaerovibrio* genera were highly influential in the separation of pig groups. The most discriminating bacterium, *Mitsuokella*, was most abundant in the PRO group (Fig. S2). This have significant implications on the functional properties in vivo of these discriminating bacterial genera; however, genomic and biochemical information remains limited for many of these discriminating genera. Future isolation and detailed characterization of these bacteria will increase our understanding of their potential roles in pig health.

A taxon‐independent (OTU‐based) analysis is a robust and appropriate method for the overall assessment of variations in the swine faecal microbiota comprising numerous unclassified bacterial phylotypes. The linear discriminant analysis of the variables (bacterial OTUs) suggested that three unclassified bacteria were the most influential in the overall separation of the groups. Specifically, the bacterial phylotype EU259447_g may be associated with lactulose in feed in the post‐weaning diet (Fig. S2); the OTU was later identified as the closest relative to *Eubacterium coprostanoligenes* based on the neighbour‐joining tree of closely related organisms (Fig. S4). This bacterium is a member of *Clostridium* cluster IV, which may be highly associated with fermentable carbohydrates (e.g. inulin, lactulose) (Sattler *et al*., 2014). Establishing the relationship of these species with the scope of the prebiotic, probiotic or synbiotic effects in swine physiology will increase our understanding of the functional and metabolic benefits of these feed additives.

Despite a previous in vitro study showing that lactulose can promote the growth of *E. faecium* (Mao *et al*., 2014), synbiotic administration did not result in significant proliferation of probiotic enterococci in the faecal microbiota of piglets. However, the synergistic action of the probiotic *E. faecium* and prebiotic lactulose is shown in the DAPC plot, which revealed distinct and separate clusters of microbial communities among treatments. It is possible that the effects of the synbiotic combination are complementary; however, this study detected the cumulative effects on the microbial composition (i.e. increased α–diversity, decreased pathogenic bacteria and increased lactobacilli population).

In conclusion, the synbiotic combination of lactulose and *E. faecium* NCIMB 11181 generated differences in the gut microbiota compared the individual effects of the prebiotic or probiotic. In other words, individual materials and their combination can lead to different results in the distal gut microbiota through independent or synergistic mechanisms. Further understanding of gut microbiota changes based on the administration of eubiotics will lead to the development of AGP‐alternatives and improve our approaches and strategies for environment‐ and animal‐friendly farming practices.

## Experimental procedures

### Animal and sample collection

All animal protocols used in this study were approved by the Dankook University Animal Care Committee. Seventy‐nine healthy piglets raised on a farm (Cheonan, Korea) were selected randomly and allocated into control and treatment groups. All piglets were born from different sows on the same day and were weaned at 4 weeks of age. Following weaning, all piglets were given the same basal feed for the next 2 weeks (Table S3) without the administration of antibiotics or feed additives. At 6 weeks, piglets were grouped into the following feeding treatments: control (CON, *n* = 15), prebiotic lactulose (LAC, *n* = 15) (Chae *et al*., 2015), probiotic *E. faecium* NCIMB 11181 (PRO, *n* = 20) (Pajarillo *et al*., 2015a) and their synbiotic combination (SYN, *n* = 29). Pigs in the CON group continued consuming the basal diet for 2‐weeks. The probiotic *E. faecium* NCIMB 11181 (Lactiferm^®^; Chr. Hansen, Nienburg, Germany) was given at a concentration of 1.0 × 10^9^ colony forming units (CFU) kg^−1^ feed, and the prebiotic lactulose was given at a concentration of 5 g kg^−1^ feed. The daily feed allotment was provided as two meals at 12‐h intervals. Animals were sheltered in an environmentally controlled room with a slatted plastic floor. Each pen was equipped with a one‐sided self‐feeder and a nipple water‐feeder for ad libitum access to feed and water throughout the experiment. The housing conditions were room temperature (25°C), 60% humidity, a mechanical ventilation system, and artificial light for 12 h using fluorescent lights.

Fresh faecal samples were collected individually from the rectum of each piglet after 2‐weeks of daily administration of prebiotic lactulose, probiotic *E. faecium* NCIMB 11181 or their synbiotic combination. Purified DNA extracts were obtained using the UltraClean Faecal DNA isolation kit (MO BIO Laboratories, Carlsbad, CA, USA) from rectal faecal grabs of individual piglets, as described previously (Pajarillo *et al*., 2014a,b). The quantity and concentration of DNA extracts were checked using the Optizen UV/Vis spectrophotometer (Mecasys, Daejeon, Korea) and sorted according to treatment group.

### 454‐pyrosequencing

PCR amplification of the DNA extracts was performed according to parameters and conditions described previously (Pajarillo *et al*., 2014a,b). PCR primers targeting the V1–V3 hypervariable regions of the bacterial 16S rRNA gene were used in this study. The PCR conditions consisted of an initial denaturing phase at 94°C for 3 min; 35 cycles of 94°C for 1 min, annealing at 55°C for 45 s, and extension at 72°C for 1 min, with a final extension at 72°C for 8 min. The visualization of PCR amplicons was performed in a 1.5% (w/v) agarose gel stained with ethidium bromide. Clear DNA amplicons visualized in agarose gels without primer dimers or contaminant bands were used in subsequent experiments. Pyrosequencing was performed using the Roche 454 GS‐FLX titanium system (454 Life Sciences, Branford, CT, USA). Raw sequence reads were processed and analysed from each faecal sample, as described previously (Jeon *et al*., 2013).

### Processing of 16S rRNA gene sequences

Downstream analysis of sequences was performed based on a previous study (Pajarillo *et al*., 2015b). The GS‐FLX pig faecal dataset for the SYN group was analysed and compared with the previous pig faecal datasets for the CON, LAC and PRO groups (Chae *et al*., 2015; Pajarillo *et al*., 2015a). Briefly, raw sequence reads generated by the 454‐pyrosequencer were demultiplexed (barcodes were removed and sequences sorted into categorical groups). Sequence reads with fewer than 300 bases were eliminated. The valid pyrosequencing reads from each pig sample was summarized in Table S4. Next, chimeras were checked and removed from the sequence data using the Bellerophon method, and sequence data were then denoized in Mothur (Schloss *et al*., 2009). The average length of high‐quality sequences without primers was 477 bp, and these sequences were used for further analysis. Using the CD‐HIT program (Li and Godzik, 2006), OTUs were assigned at a > 97% identity level. Taxonomic ranking and classification were performed using the EzTaxon database (Chun *et al*., 2007). During classification, when sequences could not be assigned into a sublevel, ‘uc’ was added to the end of the name (e.g. *Ruminococcaceae*_uc for OTUs that could be classified only at the family level). If the taxon was still unknown, the genus name was written first, and the initial letter of each unknown taxon level was written at the end of the name (e.g. if the genus name was unknown, a ‘g’ was written after the name, e.g., *Prevotella*_g; the same pattern was used for the species (s), genus (g), family (f) and accession numbers of unidentified phylotypes). The following cut‐off values were used for taxonomic assignment: species (*x* ≥ 97%), genus (97% > *x* ≥ 94%), family (94% > *x* ≥ 90%), order (90% > *x* ≥ 85%), class (85% > *x* ≥ 80%) and phylum (80% > *x* ≥ 75%), where *x* corresponds to the sequence identity between sequences within a certain OTU (Chun *et al*., 2007).

### Statistical analyses

The summaries of the percent abundances of the classified taxon groups were generated using CLCommunity software (ChunLab Inc., Seoul, Korea). Microbial richness estimates and diversity indices, including Chao1, ACE, Shannon, and Simpson (1‐D), were calculated using Mothur (version 1.32.1), with OTUs defined at the 97% identity level (Schloss *et al*., 2009). Both individual and pooled diversity indices and richness estimators are shown in the boxplot illustration (boxplot {graphics}). Differences in α‐diversity values among the CON, LAC, PRO and SYN groups were calculated at *P* < 0.05 using the Kruskal**–**Wallis test (kruskal.test {stats}).

The R software (v. 3.1.0; R Core Team, Auckland, New Zealand) was used for the following statistical and multivariate analyses. The pooled percent abundance data were imported (read.table {utils}) from CLCommunity to R software data. Differences in relative abundance among the CON, LAC, PRO and SYN groups were calculated using one‐way analysis of variance (aov {car}) for multiple independent groups and Tukey's test (TukeyHSD {car}) for the subsequent *post‐hoc* analysis. For taxon‐dependent analysis, we used 99 bacterial genera detected in at least one pig faecal sample. Metastats was employed to sort the differentially abundant genera from samples (Paulson *et al*., 2011). After removal of bacterial genera with less than 0.1% relative mean abundance, 33 differentially abundant genera with greater than 0.1% relative mean abundance remained. Next, we applied a square root (sqrt {base}) transformation to the abundance data of 33 differentially abundant bacterial genera. A heatmap (heatmap {vegan}) was generated from the square‐root‐transformed data of 33 differentially abundant genera generated above. For sample clustering analysis, we based our methods on two distance metrics: the Bray–Curtis dissimilarity matrix and the Euclidean distance, both of which were calculated in R (vegdist {vegan}). The stable algorithm used was the ‘average’ method in hierarchical clustering (hclust {stats}). The distances were calculated from 33 differentially abundant bacterial genera in each group.

For multivariate analysis of bacterial genera and bacterial OTUs (95% identity cut‐off), the adegenet package in R was used to reduce multi‐dimensionality in the multivariate framework of microbial community studies (Jombart and Ahmed, 2011). A DAPC (dapc {adegenet}) plot was constructed using a square root‐transformed data table for individual pigs from each pig group. Here, clustering of pigs was defined prior to construction of the plot based on the independent categorical variable, that is, by treatment group. Individual pig samples containing either differentially abundant bacterial genera or all bacterial OTUs (at a 95% identity cut‐off) were used to create two different DAPC plots. The principal components were selected to correspond to ≥80% cumulative variance, explained by the Eigen values of the plot, which were then subjected to linear discriminant analysis. The graphical output from the DAPC plots and canonical loading plots was then created using scatter plots (scatter {ade4}) and (loadingplot {adegenet}) respectively. The canonical loading plots were used to identify bacterial genera capable of differentiating the microbial communities according to the defined clustering groups using the user‐defined threshold (0.05) (Pajarillo *et al*., 2014b). The normalized abundance of the discriminating variables (i.e. bacterial genera, OTU) were compared among groups using a boxplot. For the phylogenetic tree reconstruction of the three most discriminating bacterial OTUs, ClustalX (version 2.1) was used to align the 16S rRNA gene sequences with other known bacterial phylotypes from the Ribosomal Database Project (RDP) Naïve Bayesian Classifier (version 2.10) using default parameters. Next, phylogenetic trees were constructed using the neighbour‐joining method in MEGA5 (Tamura *et al*., 2011; Pajarillo *et al*., 2014a). The stability of the nodes was tested by bootstrap analysis using the adjusted values of 1000 replicates.

### Data availability

All standard flowgram format (.sff) files (*n* = 79) generated by the 454‐pyrosequencer containing all raw sequence reads have been deposited at the National Center for Biotechnology Information BioProject (accession number: PRJNA319410, http://www.ncbi.nlm.nih.gov/bioproject/319410).

## Supporting information


**Fig. S1.** Rarefaction curves of pooled pig samples with an OTU definition at 97% identity level created by using CD‐HIT in Mothur. Coloured lines depict each control (CON), prebiotic lactulose (LAC), probiotic *Enterococcus faecium* NCIMB 11181 (PRO), synbiotic (SYN) groups.
