# Supplementary material for: Revealing the combined effects of lactulose and probiotic enterococci on the swine faecal microbiota using 454 pyrosequencing
Source: Microb Biotechnol. 2016 Jun 16;9(4):486–95. doi: 10.1111/1751-7915.12370 (PMC4919990; doi:10.1111/1751-7915.12370)
Supplement: Supplementary file 1 — Fig. S1. Rarefaction curves of pooled pig samples with an OTU definition at 97% identity level created by using CD‐HIT in Mothur. Coloured lines depict each control (CON), prebiotic lactulose (LAC), probiotic Enterococcus faecium NCIMB 11181 (PRO), synbiotic (SYN) groups. [file MBT2-9-486-s001.pdf]

Supporting information

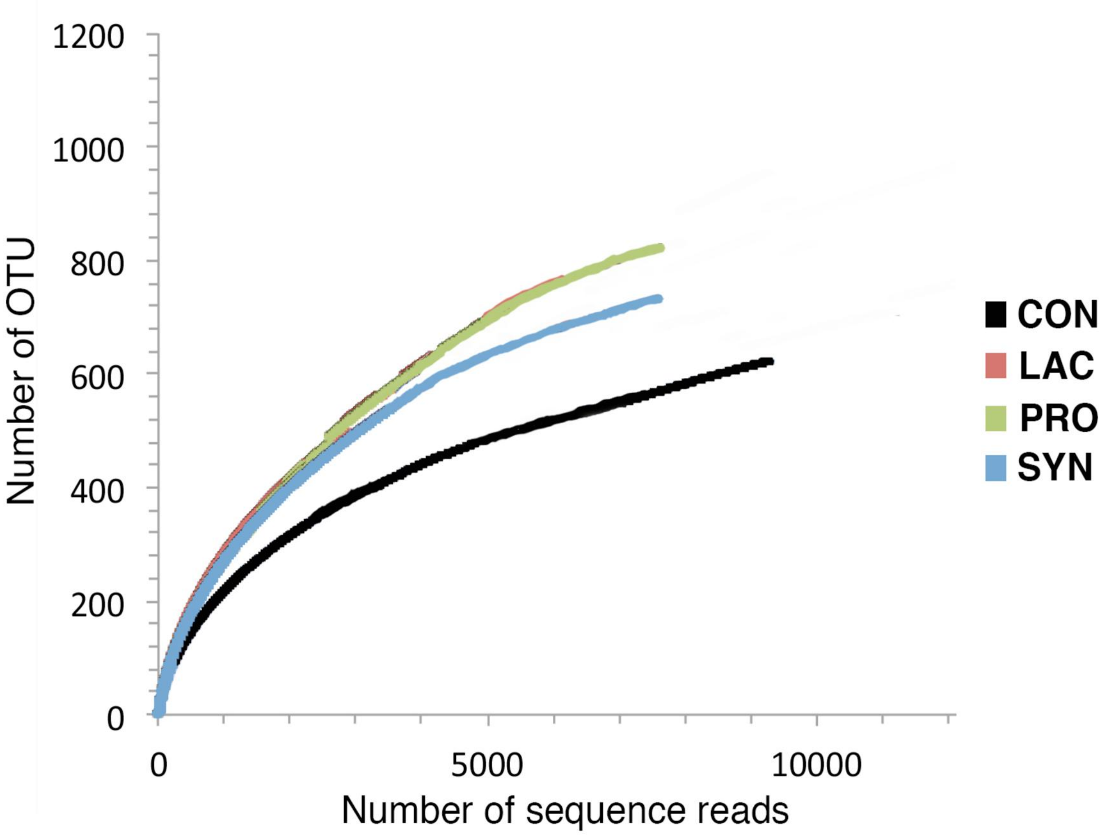

Fig. S1. Rarefaction curves of pooled pig samples with an OTU definition at 97% identity level created by using CD-HIT in Mothur. Coloured lines depict each control (CON), prebiotic lactulose (LAC), probiotic *Enterococcus faecium* NCIMB 11181 (PRO), synbiotic (SYN) groups.

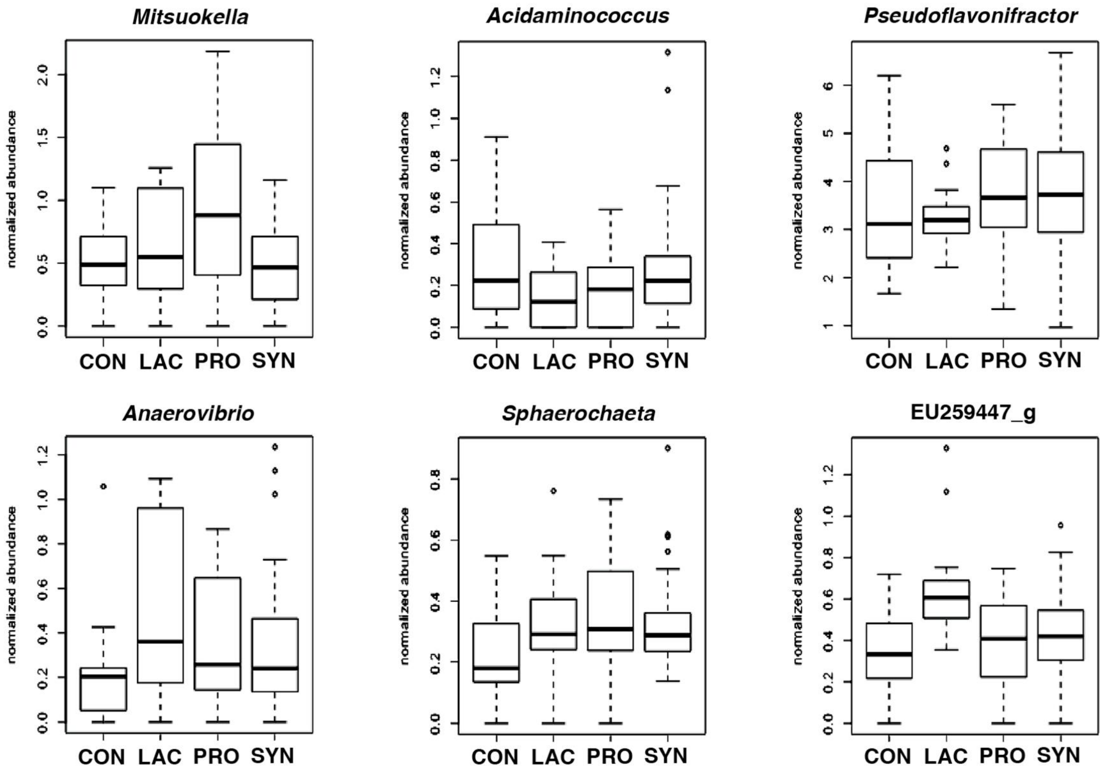

Fig. S2. Boxplot of the normalised abundance of discriminating bacterial genera and OTU according to linear discriminant analysis (loadingplot).

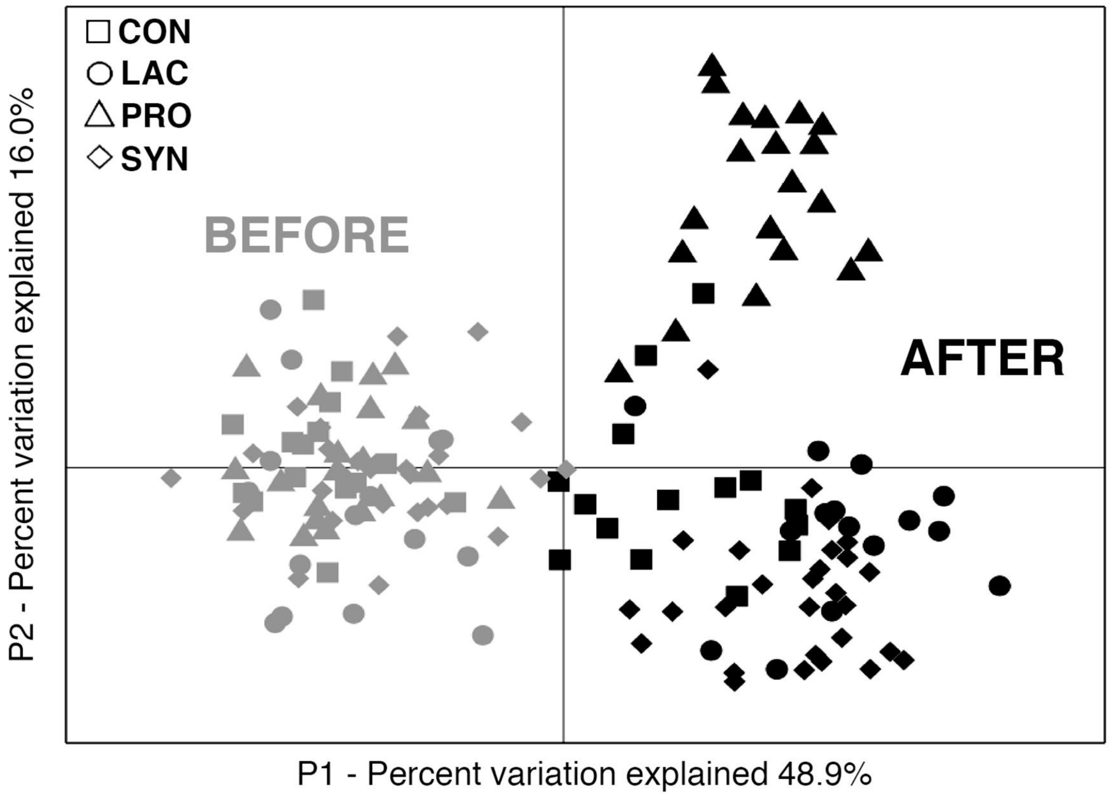

Fig. S3. Clustering of the faecal microbiota of the weaned piglets before (grey) and after (black) the treatment. The DAPC plot was created using the 33 diferentially abundant bacterial genera as variables. All pig samples (*n* = 79) belonging to control (CON), prebiotic lactulose (LAC), probiotic *E. faecium* NCIMB 11181 (PRO) and synbiotic (SYN) groups are depicted as symbols (square, circle, triangle and diamond), respectively.

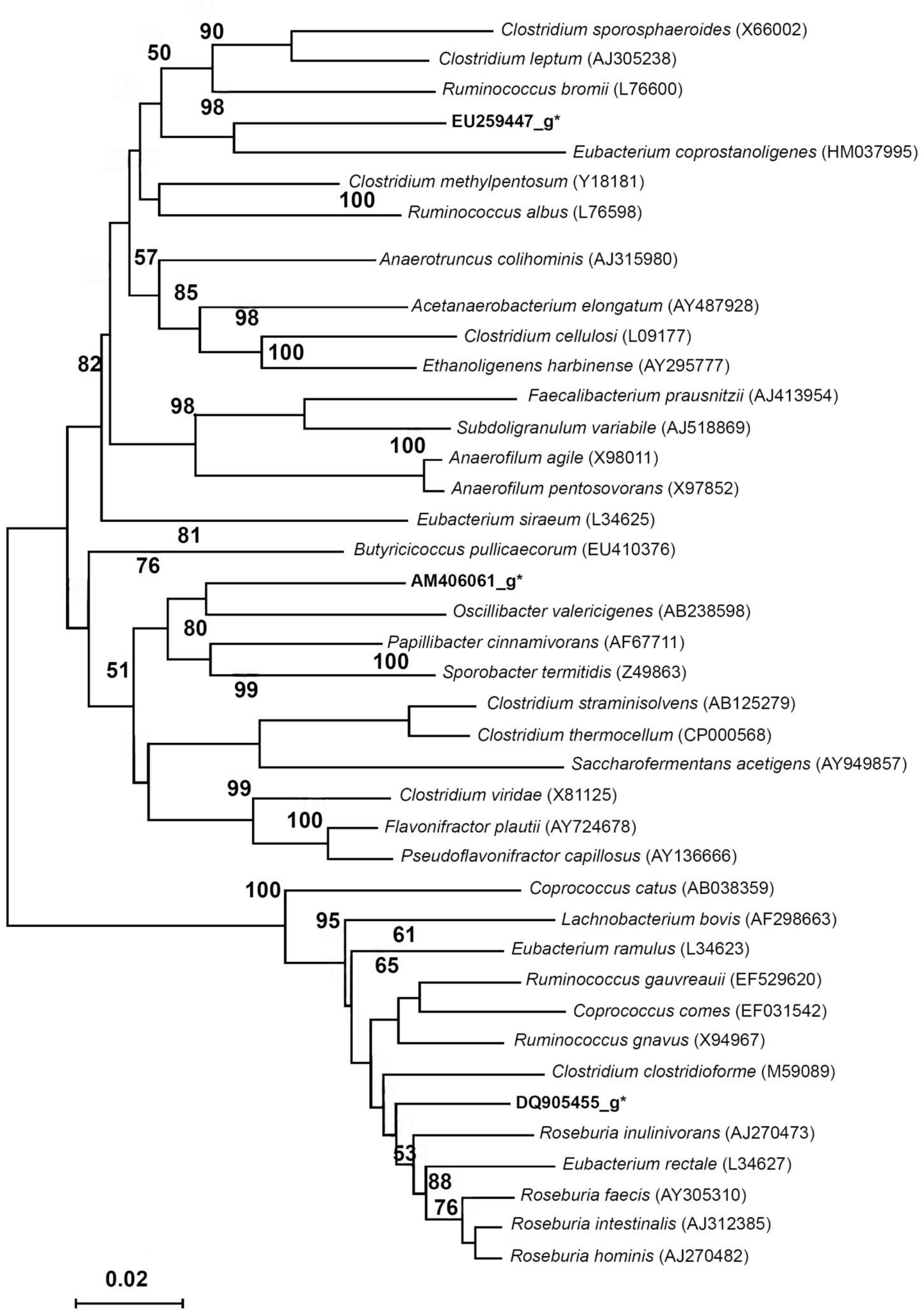

Fig. S4. Neighbour-joining phylogenetic tree showing the relationships of several bacterial strains related to three uncultured bacterial clones (denoted with asterisks). The number adjacent to each node indicates the percentage of trees having this particular configuration during 1000 bootstrap trials. The scale bar represents fixed nucleotide substitutions per sequence position. The GenBank accession numbers of the nucleotide sequences used in this analysis are provided in parentheses.

Table S1. Summary of sequence reads after quality control and microbial diversity of each group of pigs.

	CON (n = 15)	LAC (n = 15)	PRO (n = 20)	SYN (n = 29)
**Total number of reads**	80,251	100,172	98,156	208,660
**Number of OTU**[a]	669	735	853	897
**Chao1**[a]	810	1472	1376	1335
**ACE**[a]	1067	1992	1744	1740
**Shannon**[a]	4.51 (4.02, 5.15)	5.06 (4.24, 5.57)	5.21 (4.09, 5.68)	5.23 (3.74, 5.73)
**Simpson (1-D)**[a]	0.958 (0.890, 0.985)	0.977 (0.958, 0.989)	0.980 (0.922, 0.990)	0.979 (0.893, 0.991)

OTU, operational taxonomic units, ACE – abundance-based coverage estimator.
[a]Calculations were generated using CD-HIT with an OTU definition between 97% and 100% identity cut-off. The median values represent non-parametric statistical measurements. All parentheses depict the range of values from lowest to highest in each group.

Table S2. Mean values of the relative abundances (% of total sequences) of selected bacterial phyla and families between CON, LAC, PRO, SYN groups.

Bacterial taxa[a]	CON (n = 15)	LAC (n = 15)	PRO (n = 20)	SYN (n = 29)
**Phylum level[b]**				
Firmicutes	56.6	71.0^{+}	63.1	58.1^{+}
Bacteroidetes	29.4	24.4^{+}	30.0	34.3^{+}
Proteobacteria	10.4	1.33	3.01	3.98
Tenericutes	2.74	2.33	2.60	2.23
Other phyla	0.86	0.94	1.29	1.39
Firmicutes:Bacteroidetes ratio	2.47	3.39^{+}	2.35	1.99^{+}
**Family level**				
Ruminococcaceae	28.4	32.4	31.0	25.1
Prevotellaceae	16.0	12.5	17.7	18.9
Enterobacteriaceae	10.1	0.99	2.35	3.57
Veillonellaceae	8.87	6.31	6.00	4.75
Lactobacillaceae	5.67	9.20	7.97	13.8
Lachnospiraceae	5.17	7.07	5.78	6.26
Other families	25.79	31.53	29.2	27.62

[a] Mean relative abundance were calculated

[b] One-way Analysis of Variance (ANOVA) test of significance was performed to assess the difference in the percent abundance values among pig groups. A subsequent TukeyHSD post-hoc analysis was performed to check which two independent groups are significantly different. *P*-values < 0.05 are marked ($^{+}$)

Table S3. Composition of the basal (feed) diet (as-fed basis)[3]

Composition	Value
**Ingredients (g/kg)**	
Corn	553.8
Soybean meal	334.3
Molasses	25.0
Animal Fat	53.3
Difluorinated phosphate	19.3
Limestone	7.8
L-lysine HCl	1.7
Trace mineral premix[1]	1.0
Vitamin premix[2]	1.2
Salt	2.0
DL-methionine	0.3
Choline chloride	0.3
**Chemical composition (kcal/kg)**	
ME (kcal/kg)	3,360
Crude protein (%)	20.00
Lysine (%)	1.30
Calcium (%)	0.90
Phosphorus (%)	0.76
**Analysed composition**	
GE (kcal/kg)	4,112
Crude protein (%)	19.65
Calcium (%)	0.87
Phosphorus (%)	0.76

[1] Provided per kg of complete diet: 12.5 mg Mn, 179 mg Zn, 140 mg Cu, 0.5 mg I and 0.4 mg Se.

[2] Provided per kg of complete diet: 20,000 IU of vitamin A; 4,000 IU of vitamin D3; 80 IU of vitamin E; 16 mg of vitamin K3; 4 mg of thiamine, 20 mg of riboflavin; 6 mg of pyridoxine; 0.08 mg of vitamin B12; 120 mg of niacin; 50 mg of Ca-pantothenate; 2mg of folic acid and 0.08 mg of biotin.

[3] The additive was included in the diets by replacing the same amount of corn.

Table S4. Summary of the 16S rRNA gene pyrosequencing reads from each pig sample after the administration of prebiotic lactulose (LAC), probiotic *Enterococcus faecium* NCIMB 11181 (PRO) and synbiotic mixture (SYN).

Pig (Group)	Valid reads	Pig (Group)	Valid reads	Pig (Group)	Valid reads	Pig (Group)	Valid reads
C-1 (CON)	4994	L-1 (LAC)	2312	P-1 (PRO)	3730	S-1 (SYN)	3541
C-2 (CON)	3722	L-2 (LAC)	1584	P-2 (PRO)	2833	S-2 (SYN)	4916
C-3 (CON)	3086	L-3 (LAC)	2593	P-3 (PRO)	6562	S-3 (SYN)	8915
C-4 (CON)	5530	L-4 (LAC)	15793	P-4 (PRO)	9272	S-4 (SYN)	8595
C-5 (CON)	5982	L-5 (LAC)	4313	P-5 (PRO)	2445	S-5 (SYN)	6019
C-6 (CON)	2319	L-6 (LAC)	6225	P-6 (PRO)	7941	S-6 (SYN)	14385
C-7 (CON)	2509	L-7 (LAC)	6717	P-7 (PRO)	5189	S-7 (SYN)	13215
C-8 (CON)	9296	L-8 (LAC)	4960	P-8 (PRO)	4694	S-8 (SYN)	11174
C-9 (CON)	4317	L-9 (LAC)	7483	P-9 (PRO)	5102	S-9 (SYN)	9440
C-10 (CON)	4790	L-10 (LAC)	9210	P-10 (PRO)	5068	S-10 (SYN)	8093
C-11 (CON)	5384	L-11 (LAC)	9636	P-11 (PRO)	3581	S-11 (SYN)	4553
C-12 (CON)	3305	L-12 (LAC)	7132	P-12 (PRO)	4988	S-12 (SYN)	5096
C-13 (CON)	5567	L-13 (LAC)	7401	P-13 (PRO)	2752	S-13 (SYN)	5364
C-14 (CON)	9500	L-14 (LAC)	7070	P-14 (PRO)	4206	S-14 (SYN)	5124
C-15 (CON)	9950	L-15 (LAC)	7743	P-15 (PRO)	3347	S-15 (SYN)	6783
				P-16 (PRO)	4490	S-16 (SYN)	6005
				P-17 (PRO)	5300	S-17 (SYN)	5364
				P-18 (PRO)	6635	S-18 (SYN)	4676
				P-19 (PRO)	5228	S-19 (SYN)	4046
				P-20 (PRO)	4793	S-20 (SYN)	6112
						S-21 (SYN)	5295
						S-22 (SYN)	7587
						S-23 (SYN)	6928
						S-24 (SYN)	6972
						S-25 (SYN)	6371
						S-26 (SYN)	3652
						S-27 (SYN)	5444
						S-28 (SYN)	3702
						S-29 (SYN)	21293
